# Secondary spontaneous pneumothorax in patients with sarcoma treated with Pazopanib, a case control study

**DOI:** 10.1186/s12885-018-4858-8

**Published:** 2018-10-01

**Authors:** Bruce Sabath, Hasan A Muhammad, Amulya Balagani, David E Ost, Erik Vakil, Tahreem Ahmed, Macarena R Vial, Horiana B Grosu

**Affiliations:** 1 Department of Pulmonary Medicine, Unit 1462, The University of Texas MD Anderson Cancer Center, 1515 Holcombe Blvd, Houston, TX 77030 USA; 2 Thoracic Surgery, The University of Texas MD Anderson Cancer Center, Houston, TX USA; 3 Universidad del Desarrollo Clinica Alemana de Santiago, Santiago, Chile

## Abstract

**Background:**

The tyrosine kinase inhibitor pazopanib is used for treatment of sarcoma. Recent studies have suggested that the use of pazopanib may lead to the development of pneumothorax, an unexpected adverse effect in patients with sarcoma metastatic to the chest.

**Methods:**

We conducted a retrospective case control study of patients with sarcoma with metastases to the chest with pneumothorax (cases) and without pneumothorax (controls). The control population was selected from tumor registry in a 1:4 (cases to controls) ratio. The primary outcome of interest was the association between pazopanib and pneumothorax risk in patients with sarcoma metastatic to the chest. Secondary objective was to evaluate risk factors for pneumothorax.

**Results:**

We identified 41 cases and 164 controls. Using purposeful selection method the odds of developing pneumothorax while being on pazopanib was not significant in univariate (*p* = .06) and multivariable analysis (*p* = .342). On univariate analysis risk factors of pneumothorax in patients with sarcoma were age, male sex, African American race, the presence of cavitary lung nodules/masses, and the presence of pleural-based nodules/masses. On multivariate analysis, only the presence of cavitary lung nodules/masses (*P* < .001) and the presence of pleural-based nodules/masses (*P* < .001) remained as risk factors for developing pneumothorax.

**Conclusion:**

Pazopanib does not increase the risk of pneumothorax in patients with sarcoma and evidence of metastatic disease to the chest. Presence of cavitary lung nodules/masses and the presence of pleural-based nodules/masses were found to be risk factors for pneumothorax.

## Background

Nearly all underlying lung disorders can be associated with secondary spontaneous pneumothorax (SSP), nonetheless, it is most commonly associated with emphysema, cystic fibrosis, infections, and malignancy [1]. Several studies have reported an association between metastatic osteogenic or soft tissue sarcomas with pneumothorax, especially in the setting of cytotoxic chemotherapy or radiotherapy [1, 2]. To date, however, except for a limited number of case reports and small case series, little has been published to substantiate that the incidence of SSP in soft tissue sarcoma patients is higher than that in patients with other tumors [3–12].

Pazopanib, a multitarget tyrosine kinase inhibitor, was approved in 2012 for the treatment of soft tissue sarcoma on the basis of evidence of improved progression-free survival in advanced disease [13]. Since the introduction of pazopanib, however, pneumothorax has been reported as an unexpected adverse event. A recent case series found the incidence of pneumothorax to be 14%, much higher than previous data had suggested [14]. Of interest, other studies of pazopanib prescribed for non-sarcoma cancers did not report pneumothorax as an adverse event [15–17]. Therefore, higher quality evidence is needed to investigate the question on whether pazopanib is truly a risk factor.

The primary outcome of our study was an assessment of the association between pazopanib and the occurrence of SSP in patients with sarcoma. We hypothesized that pazopanib is associated with a higher risk of SSP in patients with evidence of metastatic sarcoma to the lungs. Our secondary objective was to evaluate risk factors for SSP in patients with sarcoma.

## Methods

### Study design

This retrospective case control study was designed to obtain preliminary data about the association between pazopanib and pneumothorax in patients with sarcoma metastatic to the lung. The University of Texas MD Anderson Cancer Center Institutional Review Board approved this study (IRB protocol number PA15–0761).

### Study population

**Fig. 1:**
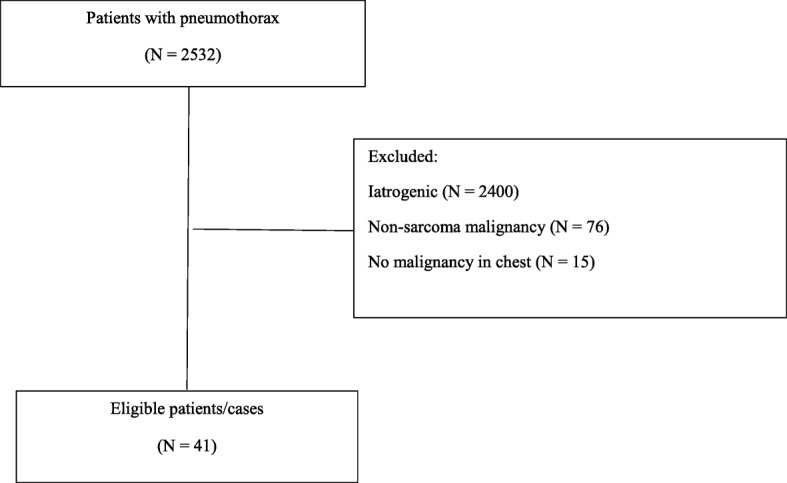
Flow chart of patient inclusion/exclusion process

All patients were 16 years of age or older and presented to our institution with a secondary spontaneous pneumothorax between January 1, 2005, and May 31, 2017. Cases were those patients with sarcoma and evidence of metastatic disease to the chest with pneumothorax. These were identified with use of the following International Classification of Diseases (ICD) diagnostic codes: ICD9 (512.89, 512.83, 512.81, 512.82, and 512.0) and ICD10 (J93, J93.9, J93.83, J93.81, J93.12, and J93.11) (Fig. 1). Controls were patients with sarcoma and evidence of metastatic disease to the chest but without pneumothorax. We identified controls from a sarcoma tumor registry at a ratio of 1:4 (cases to controls). We used density sampling to select controls. Incidence density sampling was used as controls were selected from the persons at risk (those with sarcoma) who survived at least as long as the index cases.

### Definitions

*Secondary spontaneous pneumothorax* was defined as a pneumothorax in the absence of trauma or an iatrogenic cause in a patient with evidence of metastatic disease to the chest (lung, pleura) regardless of the presence of emphysema or other risk factors for pneumothorax.

*Evidence of metastatic disease to the chest* was defined as biopsy- or cytology-proven metastatic disease, or multiple nodules/masses in a typical radiological pattern (by PET or CT) with findings sufficiently suggestive that the patient was deemed by his/her health care providers to have metastatic disease to the chest.

### Statistical considerations

For demographic and clinical characteristics, we used means and standard deviation to describe continuous variables distributed normally. We used medians and interquartile ranges (25–75%) for non-normally distributed data. We used frequencies for categorical data.

To determine whether pazopanib is associated with SSP in patients with sarcoma we used purposeful selection variables in logistic regression [18]. We used this method to improve the chances of retaining meaningful confounders, and we looked at the association of the exposure variable (pazopanib) with the other covariates and only included those variables that are associated with the exposure of interest (pazopanib) [18]. The purposeful selection process began with univariate analysis of each variable. Any variable with an arbitrary *p*-value cut-off point of 0.15 or confounding level of 15% was included in the multivariate model [18].

In a secondary analysis to determine the risk factors for SSP in patients with sarcoma we used univariate and multivariate logistic regression, with the outcome variable being SSP. Variables with a *P*-value of <.20 on univariate analysis were considered candidate variables for the multivariate regression model. Backward selection, with a *P*-value of <.05 to stay in the model, was used to arrive at a parsimonious multivariable model.

We accepted a two-tailed *P*-value of <.05 as statistically significant for all analyses. We used Intercooled Stata 13 software (College Station, TX) for data analysis.

## Results

We identified 2532 patients with a diagnosis of pneumothorax who were evaluated in our institution during the specified time period. Of these patients, 2400 patients had an iatrogenic pneumothorax, and 15 did not have metastatic disease to the chest; these were excluded. The remaining 117 patients presented with a secondary spontaneous pneumothorax associated with metastatic disease to the chest, among whom 41 had sarcoma. We identified 164 controls from institutional tumor registry. Patient’s characteristics by group was provided in Table 1.

**Table 1:** Patient characteristics

Characteristics	Cases/pneumothorax *N* = 41	Controls/no pneumothorax *N*= 164
Age, years		
Mean + SD	37.92 + 16.88	44.8 + 19.5
Gender		
Male	29 (70%)	77 (47%)
Female	12 (30%)	87 (53%)
Race		
White	21 (51%)	119 (73%)
African American	10 (24%)	14 (9%)
Hispanic/other	10 (24%)	31 (19%)
Cavitary lung nodules/masses		
Yes	26 (63%)	36 (22%)
No	15 (37%)	128 (78%)
Pleural-based lung nodules/masses		
Yes	35 (85%)	62 (38%)
No	6 (15%)	102 (62%)
Emphysema present		
Yes	2 (5%)	1 (1%)
No	39 (95%)	163 (99%)
Prior radiation to the chest		
Yes	1 (2%)	2 (2%)
No	40 (98%)	162 (98%)
Patient was receiving pazopanib		
Yes	8 (19%)	15 (9%)
No	33 (81%)	149 (91%)

The median dose of pazopanib in patients who developed pneumothorax was 800 mg (range 400 mg to 800 mg) and in those who did not develop pneumothorax was 800 mg (range 400 mg to 800 mg). This was not statistically significantly different (*p* = 0.695).

The median time on pazopanib in patients who developed pneumothorax was 96 days (range 77–1109) and in those who did not develop pneumothorax was 171 days (range 51to 584 days). This was not statistically significantly different (*p* = 0.316) .

Patient’s characteristics by exposure status with measure of the association are presented in Table 2. For the primary outcome of whether pazopanib is associated with SSP only risk factors that are associated with the confounder were included the model using purposeful selection [18]. In the univariate model the odds of developing pneumothorax while being on pazopanib was not significant [OR = 2.40 95% CI (.943 to 6.147) *p* = .06]. In the multivariate model the odds of developing pneumothorax while being on pazopanib was not significant [OR = 1.71 95% CI (.562 to 5.239) *p* = 0.342].

**Table 2:** Patient characteristics by exposure status

Characteristics	Patient was on pazopanib *N* = 23	Patient was not on pazopanib *N* = 182	*p*
Age, years			
Mean + SD	40.63 + 3.23	43. 81 + 1.45	0.456
Gender			
Male	11 (48%)	95 (52%)	
Female	12 (52%)	87 (48%)	0.693
Race			
White	18 (78%)	122 (67%)	
African American	3 (13%)	21 (12%)	
Hispanic/other	2 (9%)	39 (21%)	0.355
Cavitary lung nodules/masses			
Yes	10 (43%)	52 (29%)	
No	13 (57%)	130 (71%)	0.142
Pleural-based lung nodules/masses			
Yes	14 (61%)	83 (46%)	
No	9 (39%)	99 (54%)	0.167
Emphysema present			
Yes	0 (0%)	3 (2%)	
No	23 (100%)	179 (98%)	0.535
Prior radiation to the chest			
Yes	0 (0%)	3 (2%)	
No	23 (100%)	179 (98%)	0.535
Patient developed pneumothorax			
Yes	8 (35%)	33 (18%)	
No	15 (65%)	149 (82%)	0.060

For the secondary objective of determining the risk factors for SSP on univariate analysis, age, male sex, African American race (compared with White race), the presence of cavitary lung nodules/masses, and the presence of pleural-based nodules/masses significantly impacted the odds of developing pneumothorax (Table 2). The odds of developing pneumothorax while being treated with pazopanib did not reach statistical significance on univariate analysis (*P* = .06). In the multivariate model, only the presence of cavitary lung nodules/masses (*P* < .001) and the presence of pleural-based nodules/masses (*P* < .001) remained as risk factors for developing pneumothorax (Table 3).

**Table 3:** Risk factors for developing pneumothorax in patients with sarcoma and metastatic disease to the chest

Covariate	Univariate model				Multivariate model			
	OR	95% CI		*P*-value	OR	95% CI		*P*-value
Age	0.981	0.963	0.999	.041				
Male	2.73	1.303	5.719	.008				
Race								
White	1.000							
African American	4.047	1.589	10.307	.003				
Hispanic/Other	1.827	0.780	1.278	.164				
Cavitary lung nodule/mass	6.162	2.954	12.855	<.001	7.024	3.023	16.315	<.001
Pleural base lung nodule/mass	9.596	3.817	24.123	<.001	10.390	3.824	28.230	<.001
Emphysema present	8.358	0.739	94.54	.086				
Prior radiation to the chest present	2.025	0.179	22.893	.569				
Patient was receiving pazopanib	2.408	0.943	6.147	.06				

*CI* = confidence interval, *OR* = odds ratio

## Discussion

Soft tissue sarcomas metastasize with sizeable frequency, and in nearly one-fourth of patients, the disease metastasizes to the lungs, specifically [19]. With regard to secondary spontaneous pneumothorax as a further complication, several studies have been documented over the years [3–5, 7, 8]; recently, however, the incidence of SSP increased with the introduction of pazopanib for patients with sarcoma metastatic to the lungs [9, 10, 14, 20–23]. If there is such an association, this would be problematic because pazopanib is an attractive therapeutic option, given data showing improved prognosis in advanced disease [13]. Our study addressed this question. We showed that in patients with sarcoma and lung metastasis, pazopanib does not increase the odds of developing pneumothorax.

To our knowledge, this is the largest study of sarcoma patients with lung metastases and pneumothorax. The previous literature included case reports and small series, but none used methods to assess for risk factors of statistical significance as our investigation did. We found 41 cases of sarcoma metastatic to the lungs with pneumothorax and compared them with 164 controls without pneumothorax. The dose and the duration on pazopanib was not different on those patients who developed pneumothorax vs those who did not develop pneumothorax while on pazopanib. We were unable to find an association with pazopanib to explain the difference between these two groups. Notably, the PALETTE trial, which led to the approval of pazopanib for use in sarcoma, detected only a 2% pneumothorax rate despite a large sample of sarcoma patients [13].

Similar to our analysis, other analyses have found an association of spontaneous pneumothorax with cavitary and pleural-based lung lesions in metastatic sarcoma [5]. This may provide some insight into the mechanism underlying the development of pneumothorax, which has yet to be clearly defined in this population. It has been suggested that cytotoxic agents may induce necrosis and cavitation of lung nodules, thereby increasing risk of rupture and the development of pneumothorax [24]. Indeed, aside from pazopanib, other chemotherapeutic agents have been reported in association with pneumothorax in patients with advanced sarcoma [3–5, 7, 8].

A review of the literature describing 153 cases of spontaneous pneumothorax in patients with sarcoma noted that the drugs used most commonly before occurrence of pneumothorax included doxorubicin (49.1%), cyclophosphamide (37.6%), and vincristine (35.8%) [5]. Despite this theory, two-thirds of patients in this series developed pneumothorax before initiation of any treatment. Similarly, of the 41 study cases from our cohort, 26 (63%) had evidence of cavitation; among this group, only 6 (23%) developed cavitation while receiving pazopanib and 20 (77%) developed cavitation without receiving pazopanib. As such, it is likely the nature of the disease itself—including specific characteristics of lung metastases (e.g., cavitation, pleural location)—that increases the risk of pneumothorax rather than any agent.

Pazopanib is an antiangiogenic agent that targets the vascular endothelial growth factor receptor that in theory has the potential of causing cavitation of lung lesions and increased risk of pneumothorax [13, 25, 26]. However, our data do not support this theory since among our cases, 26 patients (63%) had evidence of cavitation, 6 (23%) of whom developed cavitation while receiving pazopanib and 20 (77%) of whom developed cavitation without receiving pazopanib. We believe that it is likely the nature of the disease, not the pazopanib that increases the risk of pneumothorax in patients with sarcoma and evidence of metastatic disease to the chest. This is also supported by the fact that other tumor types, such as renal cell or ovarian carcinomas, on pazopanib treatment did not report pneumothorax as a complication [27, 28].

We recognize several limitations to our study including those inherent to case control analyses that are subject to selection bias. The information about exposure is subject to observation bias as well. Moreover, our patients are not directly comparable to those without evidence of metastatic disease to the chest or of a different tumor type so our data cannot be generalizable to other patient populations. In addition, the small sample size may be responsible for failure for find a difference between the pneumothorax and pazopanib. We used purposeful selection method as, in addition to significant covariates, this methodology retains important confounding variables, with an attempt to develop a slightly richer model [18].

## Conclusion

On the basis of our findings we conclude that pazopanib does not increase the pneumothorax risk in patients with sarcoma and evidence of metastatic disease to the chest however the study may have been underpowered to detect a difference. Other factors, however, such as the presence of cavitary or pleural-based nodules/masses, were found to be associated with increased risk of pneumothorax in this patient population.
